# Supplementary material for: pKa Determination of a Histidine Residue in a Short Peptide Using Raman Spectroscopy
Source: Molecules. 2019 Jan 23;24(3):405. doi: 10.3390/molecules24030405 (PMC6385126; doi:10.3390/molecules24030405)
Supplement: Supplementary file 1 [file molecules-24-00405-s001.pdf]

Supplemental Information: pKa Determination of a Histidine Residue in a Peptide Using the Raman C-D Stretch

Brett H. Pogostin[1,2], Anders Malmendal[2], Casey Londergan[1*], and Karin S. Åkerfeldt[1*]

[1] Department of Chemistry, Haverford College, Haverford, PA 19041, USA; bpogostin@gmail.com (B.H.P.)
[2] Biochemistry and Structural Biology, Department of Chemistry, Lund University, PO Box 124, SE-221 00 Lund, Sweden; malmendal@gmail.com (A.M.)
* Correspondence: clonderg@haverford.edu (C.H.L.), kakerfel@haverford.edu (K.S.Å.)

Contents:

Dose-response equation S-2

Figure S1: pH-dependent Raman spectra near 1115 cm^{-1} S-3

Figure S2: pH titration fit for C4-N3 stretching region S-4

Figure S3: Difference spectrum construction for C2-D region S-4

Figure S4: Mass spectra of non-deuterated and deuterated HVD S-5

Figure 5: CW Raman spectrometer box diagram S-6

Supplementary Equations

The difference spectra in Figure 4 were overlaid, and the maximum peak intensity at 2362 cm^{-1} of each difference spectrum was plotted versus pH. This data was then fit to a sigmoidal dose-response curve (Equation 1). The pKa was extracted from the data by finding the midpoint ($Logx_0$) of this sigmoidal curve from its equation. In the case of Figure 5, the resulting sigmoid fit is shown by Equation S1.

$$y = 18282.4841 + \frac{235463.21863}{1+10^{(6.82427-x)*1.12812}} \qquad \text{(S1)}$$

The same equation was used for subsequent fits to the NMR data in Figure 6 and alternate-region Raman spectrum (Figure S2, below).

Supplementary Figures

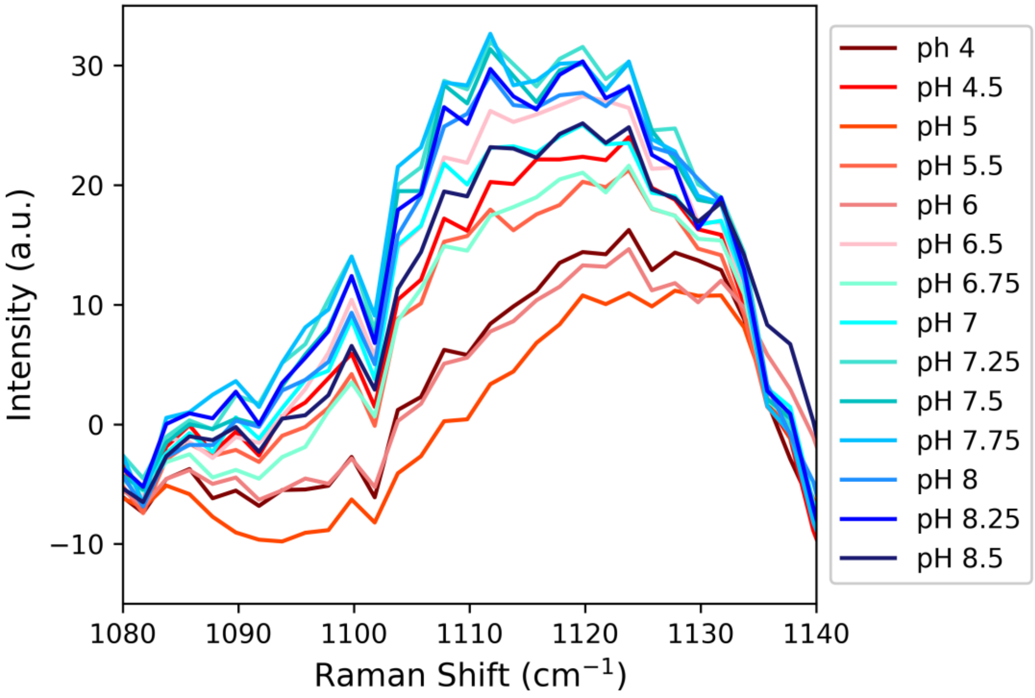

Figure S1. pH-dependent Raman spectra of H(C2-D)VD in the region of the C4-N3 stretch near 1115 cm^{-1}. The C4-N3 stretch does appear to change with pH, but not in a titratable manner. This signal was therefore found not to be useful for determining the pKa of His in the tripeptide HVD.

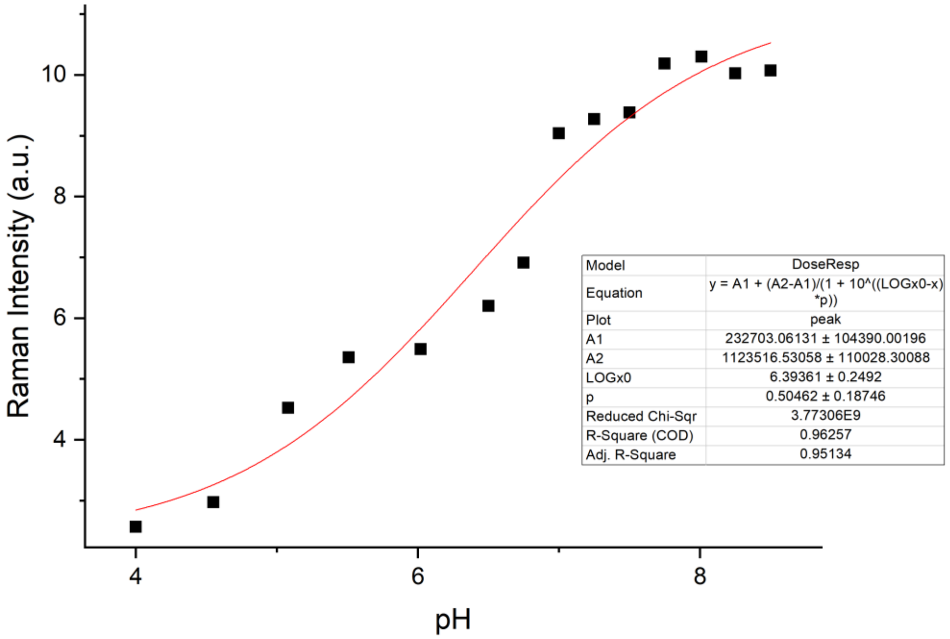

Figure S2. The maximum Raman intensity from the C4-C5 stretch at 1580 cm^{-1} (Figure 7) vs pH. A dose-response curve was then attempted to fit the data; however, the curve fits the data poorly and the pKa (6.39 ± 0.25) is reported with error margins of error and does not match the value reliably determined by ^{1}H NMR spectroscopy.

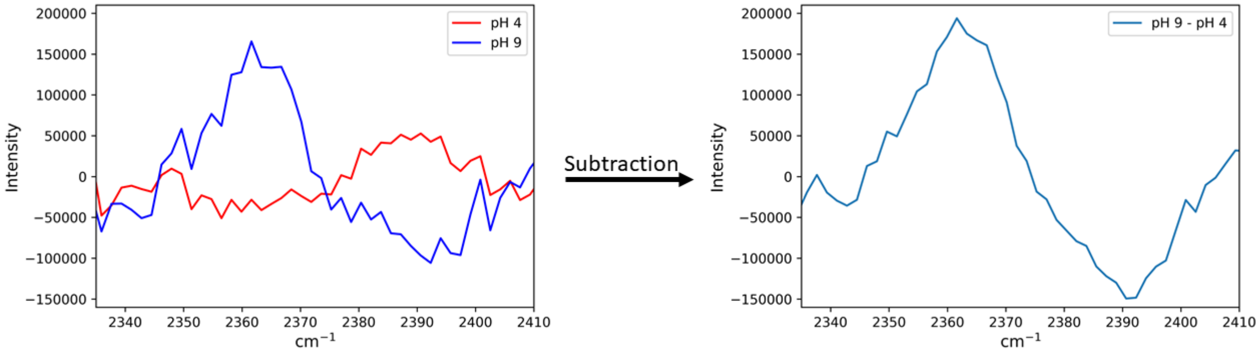

Figure S3. Difference spectra were produced by subtracting the Raman trace of a titration point (blue curve, left) from the curve from an acidic standard pH 4 sample (red curve, left). This resulted in a wave like pattern difference spectrum that displayed the disparity in peak heights between the two samples (right).

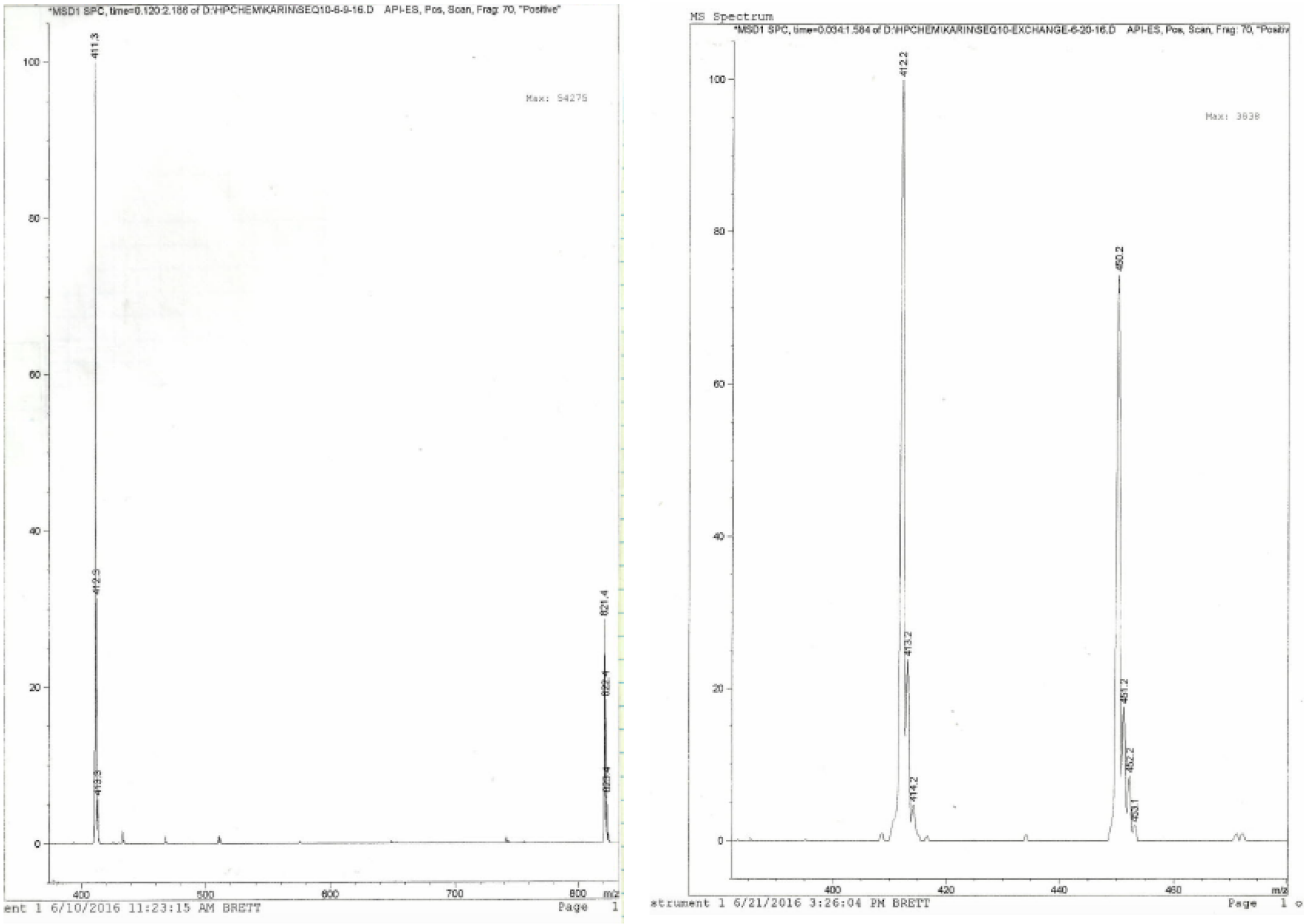

Figure S4. ES MS spectra of HVD (left) $[M+H]^+$ *m/z* = 411.3 (found), $[M+H]^+$ *m/z* = 411.2; $[2M+H]^+$ *m/z* = 821.4 (found), $[2M+H]^+$ *m/z* = 821.4 (expected); and H(C2-D)VD (right) $[M+H]^+$ *m/z* = 412.2 (found), $[M+H]^+$ *m/z* = 412.2; $[M+K]^+$ *m/z* = 450.2 (found), $[2M+H]^+$ *m/z* = 450.2.

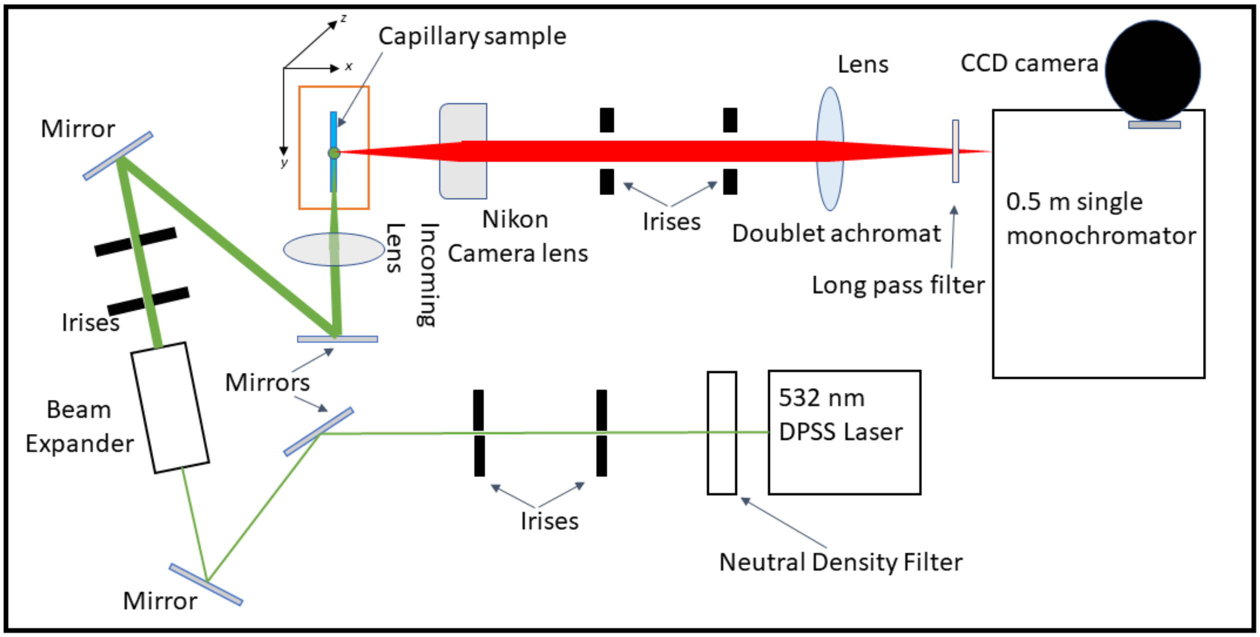

Figure S5. Box diagram of the continuous wave Raman spectrometer set-up in the Londergan lab at Haverford College.
